# Walleye Autochthonous Bacteria as Promising Probiotic Candidates against *Flavobacterium columnare*

**DOI:** 10.3389/fmicb.2017.01349

**Published:** 2017-07-18

**Authors:** Hamza Seghouani, Carlos-Enrique Garcia-Rangel, Jérémie Füller, Jeff Gauthier, Nicolas Derome

**Affiliations:** ^1^Département de Biologie, Institut de Biologie Intégrative et des Systèmes, Université Laval, Quebec QC, Canada; ^2^Centre Hospitalier de l’Université Laval, Quebec QC, Canada

**Keywords:** probiotics, *Sander vitreus*, *Flavobacterium columnare*, walleye diseases, autochthonous bacteria

## Abstract

Walleye (*Sander vitreus*) is the second most fished freshwater species in Canada. While much sought by anglers, walleye also supports substantial commercial fisheries. To cope with the recent decline of wild walleye populations, fish farmers produce juveniles for lake stocking. However, walleye breeding is particularly tedious, mostly due to high disease susceptibility at larval and juvenile developmental stages. The main threat is the columnaris disease, which is caused by *Flavobacterium columnare*, an opportunistic bacteria. As *F. columnare* strains exhibit increasing antibiotic resistance, there is a strong need to develop efficient and sustainable alternative strategies to control columnaris disease. Bacterial probiotics have been shown to mitigate infections either by enhancing host immune response or by inhibiting pathogen growth. Being successfully assessed in many fish/pathogen combinations, we developed a tailored probiotic strategy for walleye to prevent and treat columnaris disease. Thirty-seven endogenous bacterial strains were isolated from healthy walleye’s skin and gut, were tested *in vitro* against *F. columnare*. Significant antagonistic effect against *F. columnare* was measured for 2 out of 37 endogenous strains. These two probiotic strains were identified as *Pseudomonas fluorescens.* The antagonistic effect of these two successful probiotics was further validated *in vivo* during a 2-month stress trial: groups receiving probiotic treatments showed on average 53.74% survival improvement.

## Introduction

Walleye (*Sander vitreus*) is a fecund piscivorous species usually found in moderately productive lakes. Native from North America, its geographical distribution ranges from the east of United States, to the north of St. Lawrence River in eastern Quebec, Canada (Colby et al., 1979; Wilson and Nagler, 2006). Largely known as an effective predator, walleye is one of the most economically important sport and commercial species in Canada (Bernatchez and Giroux, 1991; DFO, 2007, 2012). However, in the last decades, wild walleye populations encountered significant declines, due to overfishing (Sullivan, 2003; Hunt et al., 2011). To cope with the rarefaction of this species, fish farmers started producing juveniles for lake stocking. Noteworthy, walleye breeding is particularly tedious, because optimal rearing conditions are still very challenging, mostly in terms of nutrition (Huntingford, 2004) but also because of high disease susceptibility at early developmental stages (Suomalainen et al., 2005). The most prevalent threat is the columnaris disease, which is mainly caused by *Flavobacterium columnare*, that naturally inhabits both fish microbiota and in environmental microbial communities.

*Flavobacterium columnare* is described as one of the most important bacterial diseases of freshwater fish species (Arias et al., 2004), affecting wild and cultured fish [e.g., Arctic charr, *Salvelinus alpinus* (L.), Perch, *Perca* sp. (L.), Atlantic salmon, *Salmo salar* (L.); Austin and Austin, 2007]. For instance, highly virulent strain of *F. columnare* was able to trigger death within 24 h in coho salmon fry, *Oncorhynchus kisutch* (Walbaum) (Rucker et al., 1953; see in Austin and Austin, 2007). Several studies have indicated the potential for *F. columnare* to survive for extended periods of time in water (Kunttu et al., 2009, 2012). Under laboratory conditions, *F. columnare* maintains its infectious property for more than 5 months (Kunttu et al., 2012). Welker et al. (2005) confirmed that the disease can be transmitted horizontally and indirectly through the water column without essentially being in contact fish-to-fish. When surviving outside the host, *F. columnare* inhibits virulence gene expression in order to save energy before colonizing another fish host with compromised immune system (Kunttu et al., 2009). Indeed, the occurrence of this opportunistic disease is directly related to stress, elevated temperatures, crowding, etc. (Suomalainen et al., 2005). Columnaris disease symptoms occur internally or externally (gill or skin lesions), and appear as dark-gray or yellow lesions or ulcers (Hartman, 2009). As aquaculture intensifies, overcrowding, low water quality and intensive handling increase physiological stress and physical injury, which in turn favors opportunistic pathogens (Derome et al., 2016a,b). Under such a condition, walleye larvae and juveniles become highly susceptible to columnaris disease, which causes substantial economic loss to fish farmers.

In recent decades, prevention and control of diseases in cultured animals focused research and budgets on antibiotics and chemotherapeutic agents, which are still extensively employed. To date, there are multiple evidences that intensive use antibiotics inevitably leads not only to the emergence of drug-resistant pathogens and other microorganisms, but also to the release of active molecules in the environment, both of which represent a significant risk for public health (Miranda and Zemelman, 2001; Radu et al., 2003; World Health Organization [WHO], 2014). Therefore, there is an urgent need to develop efficient and sustainable methods to control and prevent opportunistic disease such as columnariose, to meet the increasing demand for environment friendly aquaculture. Overall, such alternative methods are expected to warrant a microbiologically healthy environment to enhance fish production and economic profits (Díaz-Rosales et al., 2009). The use of probiotics to increase disease resistance and improving the overall health of terrestrial animals, has long been established as efficient, innocuous, and sustainable (Parker, 1974; Sissons, 1989; Rolfe, 2000; Scharek et al., 2007; Boutin et al., 2013; Foureaux et al., 2014; Hai, 2015). The competition between probiotic bacteria and pathogens was reported in many fish and other aquatic species (Balcázar et al., 2000, 2004, 2007). Probiotic development usually bears on two strategies: allochthonous and autochthonous. The allochthonous strategy aims to test probiotic properties of candidates that were isolated from another host organism, whereas the autochthonous strategy targets the host microbiota to isolate promising probiotic candidates (PC), in order to ensure both efficiency against the pathogen and innocuity for the host. PC isolated from host associated microbial community (i.e., microbiota) have been shown to be efficient in fish and other vertebrates such as Solea, *Solea senegalensis* (Kaup) (García de La Banda et al., 2010), Brook trout, *Salvelinus fontinalis* (Mitchill) (Boutin et al., 2012, 2013), Zebrafish, *Danio rerio* (Hamilton) (Rane and Markad, 2015), and Pigs (Hou et al., 2015). Indeed, the host microbiota, which is composed with numerous microbial strains that closely interact with each other, is a dynamic system that evolves through fish development (reviewed in Llewellyn et al., 2014; Zac Stephens et al., 2015). It is now widely acknowledged that resident bacteria contribute to host disease resistance *via* two kind of mechanisms: (1) specifically targeting pathogens either by nutritional competition, synthesis of antimicrobial compounds, or competitive exclusion from epithelial surfaces (Bermudez-Brito et al., 2012; Kamada et al., 2013); (2) mechanisms targeting the host immune signaling pathways control (Kamada et al., 2013). Regarding resistance against columnaris and other skin diseases in fish, skin mucus is playing a major role as a physical and chemical barrier (Rottmann et al., 1992). More specifically, skin microbiota associated strains have been reported to protect their host against pathogens by competitive action for adhesion sites (Vine et al., 2004; Chabrillón et al., 2005; Boutin et al., 2013; Ige, 2013), production of organic acids and other antimicrobial compounds such as bacteriocins and siderophores (Yan et al., 2002). Therefore, autochthonous skin bacteria are relevant targets to develop efficient probiotic strains against opportunistic skin disease such as columnaris. Also, as gut microbiota is a reservoir of numerous bacterial symbionts that were proved to be efficient against opportunistic pathogens (reviewed in Gomez et al., 2013), and more specifically against *Flavobacterium* (Burbank et al., 2012; Ghosh et al., 2016), those bacterial strains were also considered as PC in this work.

The goal of the present study was to develop an autochthonous probiotic strategy against columnaris disease in walleye. To do so, 37 bacterial candidates were isolated from healthy adult walleye skin and gut microbiomes in order to screen *in vitro* their antagonistic properties vis-à-vis *F. columnare*. The two candidates that demonstrated highest efficiency against *F. columnare* were further validated *in vivo* to assess both their innocuity and ability to decrease mortality rates in walleye during a stress trial.

## Materials and Methods

### *In Vitro* Experiments

#### Walleye Bacteria Sampling

Autochthonous bacteria were isolated from both skin and gut of healthy walleye (*S. vitreus*) from the Station Aquicole des Trois-Lacs (Asbestos, QC, Canada). Skin mucus samples were recovered by scraping the skin surface between the opercula and the caudal fin with a sterile razor blade. Gut mucus samples were recovered by scraping the intestine epithelial layer with a sterile Q-tip. Then, skin and gut mucus samples were diluted and homogenized 1:9 with sterile phosphate-buffered saline (1×, pH 7.4). Both pathogen and potential PC were grown on the same general growth media, i.e., Anacker and Ordal know as AO (Anacker and Ordal, 1959). The mucus dilutions were spread on fresh growth media (AO) by single-step streaking with a sterile inoculating loop. Agar plates were incubated at 20°C for 48–72 h. Individual colonies were sampled with an inoculating loop and streaked in three steps on the corresponding fresh growth media, and incubated as described above, and then stored at 4°C as solid pre-cultures. Bacterial stock cultures were prepared from pure solid culture by resuspending bacteria in excess in liquid growth medium supplemented with 15% w/v glycerol, and by storing immediately at -80°C.

#### Screening of Antagonistic Bacteria with Agar Diffusion Assays

The 37 autochthonous PC were screened on the basis of antagonism against *F. columnare* strain by diffusion of antimicrobial compounds through agar using same growth media (AO). In aseptic conditions, liquid cultures of *F. columnare* were prepared by resuspending bacteria from a solid culture in liquid AO medium up to an optical density at 600 nm (OD_600_) of 0.67. Liquid PC cultures were also prepared in a similar manner. Bacterial lawns of PC and *F. columnare* were prepared by streaking the whole surface of fresh agar media with a sterile cotton swab dipped in liquid bacterial strain culture. Before incubation, sections with a radius of 03 mm was excised from PC solid cultures and laid equidistantly upside down on *F. columnare* solid cultures. Wells were then incubated at 17°C. Inhibition surfaces around the wells were measured by scanning individually each day over a 9-day time course at a resolution of 23.6 pixels per mm, then the inhibition surfaces around the PC sections were measured using the image processing software ImageJ (NCBI, NIH)^1^. The radiuses were measured in the same manner using ImageJ. To obtain the final inhibition surface, the PC section area was subtracted from the inhibition area (Dheilly, 2014). Then, the PC showing inhibition radius were selected for *in vivo* experiment. All manipulations were executed in triplicates (Sharon et al., 2011).

### Bacterial Strain Identification

The best two PC were identified to the genus level by sequencing the 16S rRNA gene. After DNA isolation using the Dneasy Blood and Tissue Kit (Qiagen), polymerase chain reaction (PCR) amplification of the 16S rRNA gene was undertaken using the universal set of bacterial primers 331F (5′-TCCTACGGGAGGCAGCAGT-3′) (Nadkarni et al., 2002) and 1389R (5′-AGGCCCGGGAACGTATTCAC-3′) (Woo et al., 2001). PCRs were conducted in volume of 50 μL using a Biometra T1 Thermocycler, using a following amplification conditions: initial denaturation at 94°C for 2 min followed by 30 cycles of 94°C for 30 s, 55°C 1 min, 72°C for 1 min 30 s and a final extension step at 72°C for 10 min. Gel electrophoresis [2% (w/v) agarose, 100 V] was used to visualize the PCR products. Fragments were sequenced using the Big Dye Terminator V3 chemistry on an ABI 3130XL sequencer (Applied Biosystems, Foster City, United States) at the Plate-forme d’Analyses Génomiques (IBIS, Université Laval, Quebec, Canada).

### *In Vivo* Experiment

Walleye juveniles (2 cm, ∼1 g) were obtained from the Station Aquicole des Trois-Lacs (Asbestos, QC, Canada). Upon arrival, fish were acclimated in 1 m^3^ indoor tanks for 2 months. All fish were held under natural photoperiod conditions, constant temperature of 21°C, and fed daily with commercial fish food (Corey Aquafeeds). After acclimation, a total number of 324 fishes were distributed randomly between six independent recirculating 50 L tanks: each experimental group (PC1, PC2, control) was duplicated. Each tank was independent in terms of filtration and water recirculation using external filter (550 L/h).

It has been clearly demonstrated that in fish farms, *F. columnare* originates from environmental water, farm environment; then, handling practices are the principal cause triggering disease outbreaks (Pulkkinen et al., 2010; Kunttu et al., 2012). As physiological stress was identified as the most efficient disease triggering factor in intensive aquaculture (Iwama, 2011), our stress protocol aimed to mimic recurrent transfers occurring in farm conditions.

The intensity of thermal stress and mechanical stress were less extreme from previous studies (Nakano et al., 2014; Blanco Garcia et al., 2016). A combination of mechanical and thermal stresses was applied as follow: fish where captured and released into a 20-L bucket where temperature was 6°C below tank’s temperature. After a 10-min exposure to low temperature, fish where put back into their respective tanks. This stress protocol was repeated after each sampling.

Two PC isolated from walleye skin mucus and selected for their *in vitro* antagonistic activity were selected for this *in vivo* experiment. Probiotic formulations were administered twice a day (8 am and 8 pm), the mean count of isolate at each administration was 6.5 × 10^8^ colony forming unit (CFU). Moribund fish were collected daily and euthanized by overdose of MS-222 (250 mg/L). Then, dead fish were stored at -80°C for future analysis. All experiments were conducted at the Laboratoire de Recherche en Sciences Aquatiques (LARSA – Université Laval) and carried out in accordance with the LARSA guidelines approved by the “Comité de Protection des Animaux” (CPA).

### Detection of *F. columnare* in Fish Samples by Polymerase Chain Reaction

The detection of *F. columnare* was performed using the experimental procedure of Michel et al. (2002) with some modifications. Five muscle samples were taken directly from lesion and from different fish. Samples were then placed into microtubes containing 400 μL of distilled water. Using an electric homogenizer (Heidolph DIAX 100, Schwabach, Germany), the slurry was crushed and homogenized then treated in 40 μL of 40 mM Tris–ethylenediaminetetraacetic acid (EDTA) and 10 μL of 1% proteinase K. The mixture was incubated 20 min at 60°C and then 5 min at 100°C. After a 15 s centrifugation at 13,000 *g*, the supernatant was then stored at 4°C for PCR analysis.

As a negative control, two samples were used: healthy fish muscle and a pure culture of *F. psychrophilum* mixed with a healthy fish muscle tissue. As positive control, a pure culture of *F. columnare* mixed with a healthy fish muscle tissue. A DNeasy Blood and Tissue Kit (Qiagen) was used for DNA extraction on series of dilutions.

### Polymerase Chain Reaction

Using a species-specific primer for *F. columnare* Col-72F (5′-GAAGGAGCTTGTTCCTTT-3′) and Col-1260R (5′-GCCTACTTGCGTAGTG-3′) as describe by Triyanto et al. (1999). A PCR reaction was performed in final volume of 50 μL, using 1 μL of Q5^®^ High-Fidelity DNA Polymerase (M0491), 10 μL Q5 Reaction Buffer, 10 μL Q5 High GC Enhancer, 1 μL of dNTPs 10 mM, 2.5 μL of primers 10 mM, and 3 μL of template DNA samples. PCR conditions were applied as follows: samples denaturation 30 s at 98°C, then processed through 35 cycles consisting of 30 s at 98°C, 30 s at 58°C, 30 s at 72°C and 2 min at 72°C for final extension. The final products were visualized in UV light after electrophoresis in 2% agarose gel.

Furthermore, the resulting PCR products were sequenced using an Applied Biosystems ABI 3130XL DNA analyzer at the Plate-forme d’Analyses Génomiques (IBIS, Université Laval, Quebec, Canada).

### Statistical Analysis

Survival times were calculated as the time of experiment started, until death. Deaths and mortality were reported daily, and stratified by tanks and treatment. The mortality proportions between treatments during 60 days were compared by chi-square tests. We used Kaplan–Meier methods, log rank test to describe survival curves and Cox’s proportional hazards multivariate regressions were used to calculate the hazard ratios for the effect of treatment on mortality. All statistical analyses were performed using the software Rstudio, version 0.98.1102.

## Results

### *In Vitro* Screening against *F. columnare*

**FIGURE 1:**
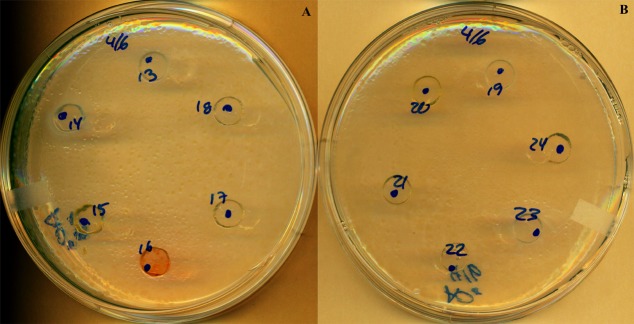
Antimicrobial activity of probiotic candidates against *F. columnare* demonstrated by the inhibition zones produced with the well-diffusion antagonism method at 216 h. PC14 **(A)**, PC23 **(B)**; all other numbers are probiotic candidates with no inhibition effects.

Among the 37 bacterial strains issued from skin mucus and the 12 bacterial strains issued from gut epithelial layer that were initially tested with agar diffusion assays against *F. columnare*, PC14 and PC23 exhibited a growth circle with an inhibition zone of respectively 5 and 3 mm diameter (**Figure 1** and **Table 1**). The plates were monitored over a 9-day period and scanned at different time (48, 120, and 216 h). The 16S rDNA gene sequence analysis showed that these two successful PC were closely related to *Pseudomonas fluorescens*, a Gram-negative bacteria, belonging to the Gammaproteobacteria subclass and shared 99% identity between each other. The closest hit in GenBank for CP14 and CP23 was *P. fluorescens* with 99.25% of average nucleotide identity (A506 complete genome accession number: NC_017911.1).

**Table 1:** Autochthonous bacteria from walleye exhibiting diffusible inhibitory effect on agar at 216 h against *F. columnare*.

Isolates	Media	Closest hit in GenBank	Percentage similarity	Sampling site	Inhibitory effect
CP14	AO	*P. fluorescens*	99.45%	Skin mucus	+++
CP23	AO	*P. fluorescens*	99.25%	Skin mucus	++


*Inhibition diameter around culture wells carved in bacterial laws of *F. columnare* at *t* = 216 h; +++, 3–5 mm wide; ++ more than 5 mm wide.*

### Antagonistic Effects against *F. columnare* in the *In Vivo* Experiment

Fish mortalities occurred within 24 h following the stress trial. Mortality events increased further until the end of the experiment for the control group. Columnaris disease symptoms were clearly identified on 40% of moribund and dead individuals.

**FIGURE 2:**
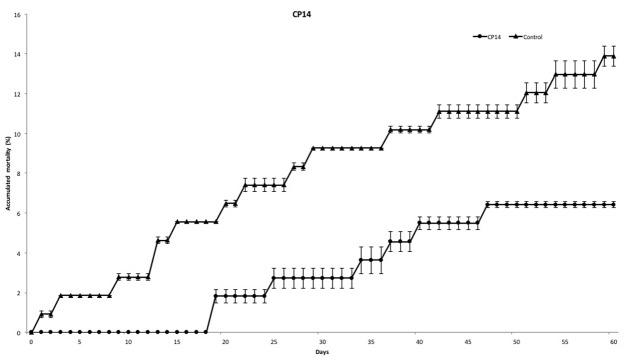
Accumulated mortality of walleye infected by *F. columnare* and treated with CP14. Probiotic culture was added to the tanks before and after stress.

**FIGURE 3:**
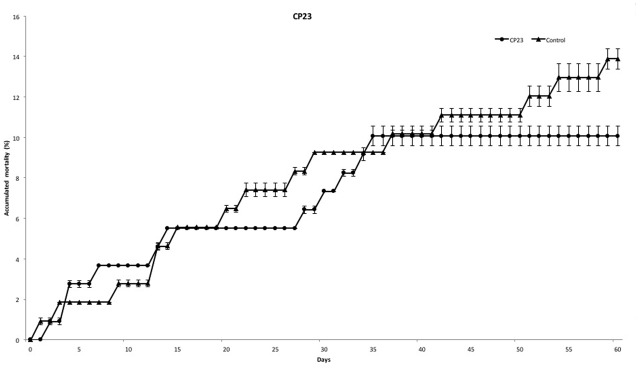
Accumulated mortality of walleye infected by *F. columnare* and treated with CP23. Probiotic culture was added to the tanks before and after stress.

**FIGURE 4:**
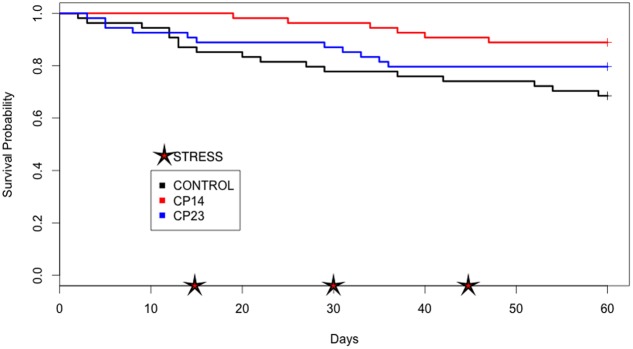
Plots of Kaplan–Meier estimate survival of control group and groups receiving probiotic (PC). Survival probability: proportion of fish that survive beyond experiment. Stress: thermal and mechanical manipulation.

At the end of experiment, mortality rate was defined for each PC. PC14 and PC23 exhibited a mean mortality rate of 6.42 and 10.07%, respectively (**Figures 2**, **3**), which was significantly lower (*p* < 0.01) for PC14 than what was observed in the control group 13.88%. There were no significant differences among duplicates for both treatment and control groups (*p* = 0.62, *p* = 0.81, and *p* = 0.72, respectively; **Figure 4**). Thus, the administration of PC14 reduced consistently and significantly the mortality across duplicates.

### PCR Analysis

The resulting PCR from the use of the specific primers Col-72F and Col-1260R was effective for all samples (**Table 2**). A band of 1200 bp was clearly identified for all samples with columnaris symptoms, whereas absent in all samples without columnaris symptoms. The sequencing of PCR products confirmed the presence of *F. columnare* in fish samples with columnaris disease symptoms (**Table 2**).

**Table 2:** PCR detection of *F. columnare* in samples.

Sample	Description	PCR detection	Percentage similarity	NCBI reference sequence
Water	Control	+	99.81	NZ_CP018912.1
Water	CP14 tank	+	99.95	NZ_CP018912.1
Water	CP23 tank	+	100.00	NC_016510.2
Muscle	Healthy fish	-	NA	NA
Muscle	Infected fish	+	100.00	NC_016510.2
Muscle	Healthy fish + *F. columnare*	+	100.00	NC_016510.2
Bacteria	*F. columnare*	+	100.00	NC_016510.2


*+, *F. columnare* detected; -, *F. columnare* not detected; percentage of similarity of the closest hit of *F. columnare* in GenBank.*

## Discussion

Fish recruit bacterial strains to build up their microbiota directly from the environmental water microbial community, however, microbiota assemblages are very specific to the corresponding body surface, and highly differentiated from environmental bacterial communities (Apun et al., 1999; Diler et al., 2000; Austin, 2006; Llewellyn et al., 2014, 2015; Zac Stephens et al., 2015; Sylvain et al., 2016). Many studies showed that during their long co-evolution, microbial communities and their hosts have established mutualistic interactions for many physiological aspects, providing major beneficial molecules and services to their host such as enzyme synthesis, vitamins, metabolites, antimicrobial compounds, immune system development, and maturation (Rawls et al., 2004; Salinas et al., 2006; Nayak, 2010; Tremaroli and Bäckhed, 2012).

The present study aimed to take advantage of beneficial host microbiota properties to develop an autochthonous probiotic strategy against columnaris disease in walleye. Among the 49 isolated strains screened *in vitro* for their potential antagonistic properties vis-à-vis *F. columnare*, two PC produced clear inhibition circles on *F. columnare* lawns (PC14 and PC23). Such result suggests their antagonistic effect against *F. columnare* is likely due, at least in part, to a diffusible antimicrobial compound. These two successful PC were further validated *in vivo* to test both their innocuity vis-à-vis *S. vitreus* and their ability to decrease mortality in a stress trial. According to our results, the antagonistic properties of these two PC strains measured *in vitro* were potentially maintained *in vivo* by improving significantly fish survival (+53%) under a context of stress *trial* which, according to both PCR analysis and sequence identification, triggered columnaris disease. However, it is not clear if the same mechanisms of action were involved in both *in vitro* and *in vivo* contexts. Overall, the successful administration of these two probiotic strains to walleye is coherent with previous studies that observed both *in vitro* and *in vivo* beneficial effects (Gram et al., 2001; Boutin et al., 2012). Gram and Ringø (2005) proposed that an effective probiotic should be identified by its capability to reduce the incidence of disease with a decrease of mortality. More recently, Boutin et al. (2012) confirmed that a positive effect of probiotic is represented by significant decrease of mortality. Still, it is premature to state whether the antibacterial properties observed *in vitro* are the sole mechanism that favored fish survival.

The two successful PC were closely related to *P. fluorescens*, belonging to the Gammaproteobacteria subclass. Interestingly, antagonistic properties against pathogenic bacteria and fungi were frequently documented in other aquatic *Pseudomonas* species and have been suggested to present a high interest as autochthonous PC for aquaculture (Sugita et al., 1996; Gram et al., 2001; Nayak, 2010). Furthermore, some authors concluded that the recurrent presence of *Pseudomonas* on fish skin represents potentially a promising probiotic strains for fish (Bly et al., 1997; Gram et al., 1999). For instance, *Pseudomonas aeruginosa* and *P. aeruginosa* YC58 improved the survival of two varieties of oysters (*Pinctada mazatlanica* and *Crassostrea corteziensis*; Aguilar-Macías et al., 2010; Campa-Cordova et al., 2011). Other *Pseudomonas* were successfully tested against different pathogenic organisms *in vitro* such as *Aeromonas hydrophila* (Eissa and El-Ghiet, 2011; Samal et al., 2014) and *Vibrio midae* (Silva-Aciares et al., 2010). Two studies showed the beneficial effect of *P. fluorescens* as a promising PC to control pathogens in two distantly related fish species: rainbow trout, *Oncorhynchus mykiss* (Walbaum) (Gram et al., 1999) and Nile tilapia, *Oreochromis niloticus* (L.) (Eissa et al., 2014). In our study, annotation of the two PC 16S rDNA partial sequence (<1000 nucleotides) indicated that both of them shared 99% of identity with *P. fluorescens* A506. This strain is registered as BlightBan^®^ A506 and has been commercialized as a microbial pest control agent against a *Erwinia amylovora*, a pathogen that affect apples and pear trees (Health Canada Pest Management Regulatory Agency, 2011).

The *in vivo* probiotic effect of PC from our study was efficient in promoting fish survival in a context of *F. columnare* disease, which occurred after fish handling and thermal stress. However, regarding the current data, it is not possible to conclude whether the important mortality decrease observed in this experiment was only due to the antibacterial effect attributed to the *Pseudomonas* strain. Indeed, the *Pseudomonas* genus encompasses numerous strains, those own diverse mechanisms of action: some are producing bioactive agents such as bacteriocins, pyocin, and phenazinen (Tinh et al., 2007), other strains are triggering bacterial cell membrane lysis, or are producing inhibitors of fatty acid synthesis pathway such as acetyl-CoA, and nitrous oxide (Freiberg et al., 2004; Isnansetyo and Kamei, 2009).

The time scale of a probiotic administration experiment and mode of supplementation are an important criterion affecting the establishment of the probiotic bacteria, their persistence, and even their influence on host immune response. Studies showed that application of probiotic directly to the rearing water play a significant role to health benefits of fish, but also to the rearing environmental (Boyd and Massaut, 1999; Zhou et al., 2010).

The significant improvement of fish survival obtained after 2 months of probiotic administration suggests that autochthonous probiotic strategy is a promising avenue in aquaculture industries. As many studies showed the effectiveness of (allochthonous/autochthonous) probiotics *in vivo* to decrease mortality and even prevent disease in many species: shrimp, *Litopenaeus vannamei* (Boone) (Kongnum and Hongpattarakere, 2012), Brook trout (Boutin et al., 2012), and Nile tilapia (Villamil et al., 2012; Eissa et al., 2014). Our work confirms further how efficiently endogenous probiotic can be developed “*de novo*” to decrease mortality in a context of fish farming industry stressing conditions. Overall, the use of endogenous probiotics in aquaculture provides a straightforward tool to both efficiently and sustainably increase survival rates in aquaculture.

## Author Contributions

HS performed *in vivo* experiment, data analysis, and writing manuscript. C-EG-R performed *in vitro* and *in vivo* experiment. JF worked with C-EG-R on *in vitro* experiment. JG brought fish and helped with *in vitro* experiment. ND revised the manuscript and supervised the work.

## Conflict of Interest Statement

The authors declare that the research was conducted in the absence of any commercial or financial relationships that could be construed as a potential conflict of interest.

## Footnotes

1 http://imagej.nih.gov/ij/

## References

[B1] Aguilar-Macías O. Ojeda-Ramírez J. Campa-Córdova A. Saucedo P. (2010). Evaluation of natural and commercial probiotics for improving growth and survival of the pearl oyster, pinctada mazatlanica, during late hatchery and early field culturing. *J. World Aquacult. Soc.* 41 447–454. 10.1111/j.1749-7345.2010.00386.x

[B2] Anacker R. L. Ordal E. J. (1959). Studies on the *Myxobacterium chondrococcus* columnaris: II, Bacteriocins. *J. Bacteriol.* 78 33–40. 13672907 10.1128/jb.78.1.33-40.1959 PMC290481

[B3] Apun K. Yusof A. Jugang K. (1999). Distribution of bacteria in tropical freshwater fish and ponds. *Int. J. Environ. Health Res.* 9 285–292. 10.1080/09603129973083

[B4] Arias C. Welker T. Shoemaker C. Abernathy J. Klesius P. (2004). Genetic fingerprinting of *Flavobacterium columnare* isolates from cultured fish. *J. Appl. Microbiol.* 97 421–428. 10.1111/j.1365-2672.2004.02314.x 15239710

[B5] Austin B. (2006). The bacterial microflora of fish, revised. *Sci. World J.* 6 931–945. 10.1100/tsw.2006.181 PMC5917212 16906326

[B6] Austin B. Austin D. A. (2007). *Bacterial Fish Pathogens Disease of Farmed and Wild Fish*, 4th Edn London: Praxis Publishing, 552.

[B7] Balcázar J. Vendrell D. de Blas I. Ruiz-Zarzuela I. Gironés O. Múzquiz J. (2007). In vitro competitive adhesion and production of antagonistic compounds by lactic acid bacteria against fish pathogens. *Vet. Microbiol.* 122 373–380. 10.1016/j.vetmic.2007.01.023 17336468

[B8] Balcázar J. L. De Blas I. Ruiz-Zarzuela I. Cunningham D. Vendrell D. Múzquiz J. L. (2000). The role of probiotics in aquaculture. *Vet. Microbiol.* 114 173–186. 10.1016/j.vetmic.2006.01.009 16490324

[B9] Balcázar J. L. Vendrell D. Blas I. D. Ruiz-Zarzuela I. Múzquiz J. L. (2004). Probiotics: a tool for the future of fish and shellfish health management. *J. Aquacult. Trop.* 19 239–242.

[B10] Bermudez-Brito M. Plaza-Diaz J. Munoz-Quezada S. Gomez-Llorente C. Gil A. (2012). Probiotic mechanisms of action. *Ann. Nutr. Metab.* 61 160–174. 10.1159/000342079 23037511

[B11] Bernatchez L. Giroux M. (1991). *Guide des Poissons D’eau Douce du Queìbec et Leur Distribution Dans l’Est du Canada*, 1st Edn La Prairie, QC: Eìditions Broquet.

[B12] Blanco Garcia A. Jansen H. M. van Bakhuizen W. van Houcke J. Smaal A. (2016). “How to measure mussel vitality: evaluation of survival in air and neutral red assay as a stress response indicator for the blue mussel (Mytilus edulis) processing industry,” in *Poster Session Presented at European Aquaculture Society*, Edinburgh.

[B13] Bly J. Quiniou S. Lawson L. Clem L. (1997). Inhibition of Saprolegnia pathogenic for fish by *Pseudomonas fluorescens*. *J. Fish Dis.* 20 35–40. 10.1046/j.1365-2761.1997.d01-104.x

[B14] Boutin S. Audet C. Derome N. (2013). Probiotic treatment by indigenous bacteria decreases mortality without disturbing the natural microbiota of *Salvelinus fontinalis*. *Can. J. Microbiol.* 59 662–670. 10.1139/cjm-2013-0443 24102219

[B15] Boutin S. Bernatchez L. Audet C. Derôme N. (2012). Antagonistic effect of indigenous skin bacteria of brook charr (*Salvelinus fontinalis*) against *Flavobacterium columnare* and *F. psychrophilum*. *Vet. Microbiol.* 155 355–361. 10.1016/j.vetmic.2011.09.002 21958747

[B16] Boyd C. E. Massaut L. (1999). Risks associated with the use of chemicals in pond aquaculture. *Aquacult. Eng.* 20 113–132. 10.1016/S0144-8609(99)00010-2

[B17] Burbank D. LaPatra S. Fornshell G. Cain K. (2012). Isolation of bacterial probiotic candidates from the gastrointestinal tract of rainbow trout, *Oncorhynchus mykiss* (walbaum), and screening for inhibitory activity against *Flavobacterium psychrophilum*. *J. Fish Dis.* 35 809–816. 10.1111/j.1365-2761.2012.01432.x 22913277

[B18] Campa-Cordova A. I. Luna-Gonzalez A. Mazon-Suastegui J. M. Aguirre-Guzman G. Ascencio F. Gonzalez-Ocampo H. A. (2011). Effect of probiotic bacteria on survival and growth of Cortez oyster larvae, *Crassostrea corteziensis* (Bivalvia: Ostreidae). *Revista de Biología Tropical* 59 183–191. 21516645

[B19] Chabrillón M. Rico R. Balebona M. Morinigo M. (2005). Adhesion to sole, *Solea senegalensis* Kaup, mucus of microorganisms isolated from farmed fish, and their interaction with *Photobacterium damselae* subsp. piscicida. *J. Fish Dis.* 28 229–237. 10.1111/j.1365-2761.2005.00623.x 15813865

[B20] Colby P. J. McNicol R. E. Ryder R. A. (1979). *Synopsis of Biological Data on the Walleye Stizostedion v. vitreum (Mitchill 1918). FAO Fisheries Synopsis No. 119*. Rome: Food and Agriculture Organization of the United Nation, 139.

[B21] Derome N. Gauthier J. Boutin S. Llewellyn M. (2016a). “Bacterial opportunistic pathogens of fish,” in *Advances in Environmental Microbiology, The Rasputin Effect: When Commensals and Symbionts Become Parasitic*, ed. Christon J. H. (Heidelberg: Springer international), 81–108. 10.1007/978-3-319-28170-4

[B22] Derome N. Gauthier J. Boutin S. Llewellyn M. (2016b). “Fungal secondary invaders of fish,” in *Advances in Environmental Microbiology, The Rasputin Effect: When Commensals and Symbionts Become Parasitic*, Chap. 5 ed. Christon J. H. (Heidelberg: Springer international), 109–126. 10.1007/978-3-319-28170-4

[B23] DFO (2007). *Survey of Recreational Fishing in Canada (2005)*. Ottawa, ON: Fisheries and Oceans Canada Statistics Division.

[B24] DFO (2012). *Survey of Recreational Fishing in Canada (2010)*. Ottawa, ON: Fisheries and Oceans Canada Statistics Division.

[B25] Dheilly N. (2014). Holobiont–holobiont interactions: redefining host–parasite interactions. *PLoS Pathog.* 10:e1004093 10.1371/journal.ppat.1004093 PMC4081813 24992663

[B26] Díaz-Rosales P. Arijo S. Chabrillón M. Alarcón F. J. Tapia-Paniagua S. T. Martínez-Manzanares E. (2009). Effects of two closely related probiotics on respiratory burst activity of Senegalese sole (*Solea senegalensis*, Kaup) phagocytes, and protection against *Photobacterium damselae* subsp. piscicida. *Aquaculture* 293 16–21. 10.1016/j.aquaculture.2009.03.050

[B27] Diler Ö Altun S. Çalikuşu F. Diler A. (2000). A study on qualitative and quantitative bacterial flora of the rainbow trout (*Oncorhynchus mykiss*) living in different fish farms. *Turkish J. Vet. Anim. Sci.* 24 251–260.

[B28] Eissa N. El-Gheit El A. Shaheen A. A. (2014). Protective effect of *Pseudomonas* fluorescens as a probiotic in controlling fish pathogens. *Am. J. Biosci.* 2 175–181. 10.11648/j.ajbio.20140205.12

[B29] Eissa N. El-Ghiet E. A. (2011). Efficacy of *Pseudomonas fluorescens* as biological control agents against *Aeromonas hydrophila* infection in Oreochromis niloticus. *World J. Fish Mar. Sci.* 3 564–569. 10.11648/j.ajbio.20140205.12

[B30] Foureaux R. Messora M. de Oliveira L. Napimoga M. Pereira A. Ferreira M. (2014). Effects of probiotic therapy on metabolic and inflammatory parameters of rats with ligature-induced periodontitis associated with restraint stress. *J. Periodontol.* 85 975–983. 10.1902/jop.2013.130356 24171503

[B31] Freiberg C. Brunner N. Schiffer G. Lampe T. Pohlmann J. Brands M. (2004). Identification and characterization of the first class of potent bacterial Acetyl-CoA carboxylase inhibitors with antibacterial activity. *J. Biol. Chem.* 279 26066–26073. 10.1074/jbc.m402989200 15066985

[B32] García de La Banda I. Lobo C. León-Rubio J. Tapia-Paniagua S. Balebona M. Moriñigo M. (2010). Influence of two closely related probiotics on juvenile Senegalese sole (*Solea senegalensis*, Kaup 1858) performance and protection against *Photobacterium damselae* subsp. piscicida. *Aquaculture* 306 281–288. 10.1016/j.aquaculture.2010.05.008

[B33] Ghosh B. Cain K. D. Nowak B. F. Bridle A. R. (2016). Microencapsulation of a putative probiotic *Enterobacter* species, C6-6, to protect rainbow trout, *Oncorhynchus mykiss* (Walbaum), against bacterial coldwater disease. *J. Fish Dis.* 39 1–11. 10.1111/jfd.12311 25272249

[B34] Gomez D. Sunyer J. Salinas I. (2013). The mucosal immune system of fish: the evolution of tolerating commensals while fighting pathogens. *Fish Shellf. Immunol.* 35 1729–1739. 10.1016/j.fsi.2013.09.032 PMC3963484 24099804

[B35] Gram L. Løvold T. Nielsen J. Melchiorsen J. Spanggaard B. (2001). In vitro antagonism of the probiont *Pseudomonas fluorescens* strain AH2 against *Aeromonas salmonicida* does not confer protection of salmon against furunculosis. *Aquaculture* 199 1–11. 10.1016/s0044-8486(01)00565-8

[B36] Gram L. Melchiorsen J. Spanggaard B. Huber I. Nielsen T. F. (1999). Inhibition of *Vibrio anguillarum* by *Pseudomonas fluorescens* AH2, a possible probiotic treatment of fish. *Appl. Environ. Microbiol.* 65 969–973. 10049849 10.1128/aem.65.3.969-973.1999 PMC91130

[B37] Gram L. Ringø E. (2005). “Prospects of fish probiotics,” in *Microbial Ecology of the Growing Animal*, eds Holzapfel W. H. Naughton P. J. (Amsterdam: Elsevier), 379–417. 10.1016/S1877-1823(09)70050-5

[B38] Hai N. V. (2015). The use of probiotics in aquaculture. *J. Appl. Microbiol.* 4 917–935. 10.1111/jam.12886 26119489

[B39] Hartman G. F. (2009). A biological synopsis of walleye (*Sander vitreus*). *Can. Manuscript Rep. Fish. Aquatic Sci.* 2888:48.

[B40] Health Canada Pest Management Regulatory Agency (2011). *ARCHIVED - Pseudomonas fluorescens Strain A506 - Proposed Registration Decision PRD2011-18 - Health Canada Consultation Document. HC-SC.GC.CA.* Available at: http://www.hc-sc.gc.ca/-spc/pest/part/consultations/_prd2011-18/prd2011-18-eng.php [accessed March 14, 2016].

[B41] Hou C. Zeng X. Yang F. Liu H. Qiao S. (2015). Study and use of the probiotic *Lactobacillus reuteri* in pigs: a review. *J. Anim. Sci. Biotechnol.* 6:14 10.1186/s40104-015-0014-3 PMC4423586 25954504

[B42] Hunt L. Arlinghaus R. Lester N. Kushneriuk R. (2011). The effects of regional angling effort, angler behavior, and harvesting efficiency on landscape patterns of overfishing. *Ecol. Appl.* 21 2555–2575. 10.1890/10-1237.1 22073644

[B43] Huntingford F. (2004). Implications of domestication and rearing conditions for the behaviour of cultivated fishes. *J. Fish Biol.* 65 122–142. 10.1111/j.0022-1112.2004.00562.x

[B44] Ige B. (2013). Probiotics use in intensive fish farming. *Afr. J. Microbiol. Res.* 7 2701–2711. 10.5897/ajmr12x.021

[B45] Isnansetyo A. Kamei Y. (2009). Bioactive substances produced by marine isolates of *Pseudomonas*. *J. Ind. Microbiol. Biotechnol.* 36 1239–1248. 10.1007/s10295-009-0611-2 19582493

[B46] Iwama G. (2011). *Fish Stress and Health in Aquaculture*, 1st Edn Cambridge: Cambridge University Press.

[B47] Kamada N. Chen G. Inohara N. Núñez G. (2013). Control of pathogens and pathobionts by the gut microbiota. *Nat. Immunol.* 14 685–690. 10.1038/ni.2608 23778796 PMC4083503

[B48] Kongnum K. Hongpattarakere T. (2012). Effect of *Lactobacillus plantarum* isolated from digestive tract of wild shrimp on growth and survival of white shrimp (*Litopenaeus vannamei*) challenged with *Vibrio harveyi*. *Fish Shellf. Immunol.* 32 170–177. 10.1016/j.fsi.2011.11.008 22126856

[B49] Kunttu H. M. Sundberg L. R. Pulkkinen K. Valtonen E. T. (2012). Environment may be the source of *Flavobacterium columnare* outbreaks at fish farms. *Environ. Microbiol. Rep.* 4 398–402. 10.1111/j.1758-2229.2012.00342.x 23760824

[B50] Kunttu H. M. Valtonen E. T. Jokinen E. I. Suomalainen L. R. (2009). Saprophytism of a fish pathogen as a transmission strategy. *Epidemics* 1 96–100. 10.1016/j.epidem.2009.04.003 21352756

[B51] Llewellyn M. Boutin S. Hoseinifar S. Derome N. (2014). Teleost microbiomes: the state of the art in their characterization, manipulation and importance in aquaculture and fisheries. *Front. Microbiol.* 5:207 10.3389/fmicb.2014.00207 PMC4040438 24917852

[B52] Llewellyn M. McGinnity P. Dionne M. Letourneau J. Thonier F. Carvalho G. (2015). The biogeography of the atlantic salmon (*Salmo salar*) gut microbiome. *ISME J.* 10 1280–1284. 10.1038/ismej.2015.189 26517698 PMC5029221

[B53] Michel C. Messiaen S. Bernardet J. (2002). Muscle infections in imported neon tetra, *Paracheirodon innesi* Myers: limited occurrence of microsporidia and predominance of severe forms of columnaris disease caused by an Asian genomovar of *Flavobacterium columnare*. *J. Fish Dis.* 25 253–263. 10.1046/j.1365-2761.2002.00364.x

[B54] Miranda C. D. Zemelman R. (2001). Antibiotic resistant bacteria in fish from the concepcion bay, Chile. *Mar. Pollut. Bull.* 42 1096–1102. 10.1016/S0025-326X(01)00093-5 11763221

[B55] Nadkarni M. Hunter N. Jacques N. Martin F. (2002). Determination of bacterial load by real-time PCR using a broad-range (universal) probe and primers set. *Microbiology* 148 257–266. 10.1099/00221287-148-1-257 11782518

[B56] Nakano T. Kameda M. Shoji Y. Hayashi S. Yamaguchi T. Sato M. (2014). Effect of severe environmental thermal stress on redox state in salmon. *Redox Biol.* 2 772–776. 10.1016/j.redox.2014.05.007 25009778 PMC4085342

[B57] Nayak S. (2010). Probiotics and immunity: a fish perspective. *Fish Shellf. Immunol.* 29 2–14. 10.1016/j.fsi.2010.02.017 20219683

[B58] Parker R. (1974). Probiotics, the other half of the antibiotic story. *Anim. Nutr. Health* 29 4–8.

[B59] Pulkkinen K. Suomalainen L. R. Read A. F. Ebert D. Rintamäki P. Valtonen E. T. (2010). Intensive fish farming and the evolution of pathogen virulence: the case of columnaris disease in Finland. *Proc. R. Soc. Lond. B Biol. Sci.* 277 593–600. 10.1098/rspb.2009.1659 PMC2842694 19864284

[B60] Radu S. Ahmad N. Ling F. H. Reezal A. (2003). Prevalence and resistance to antibiotics for Aeromonas species from retail fish in Malaysia. *Int. J. Food Microbiol.* 81 261–266. 10.1016/S0168-1605(02)00228-3 12485753

[B61] Rane M. Markad A. (2015). Effects of probiotic on the growth and survival of zebra fish (*Danio rerio*). *Int. J. Sci. Res.* 4 1839–1841. 10.1071/RD12187

[B62] Rawls J. Samuel B. Gordon J. (2004). From the cover: gnotobiotic zebrafish reveal evolutionarily conserved responses to the gut microbiota. *Proc. Natl. Acad. Sci. U.S.A.* 101 4596–4601. 10.1073/pnas.0400706101 15070763 PMC384792

[B63] Rolfe R. D. (2000). The role of probiotic cultures in the control of gastrointestinal health. *J. Nutr.* 130 396S–402S. 10721914 10.1093/jn/130.2.396S

[B64] Rucker R. R. Earp B. J. Ordal E. J. (1953). Infectious diseases of Pacific salmon. *Trans. Am. Fish. Soc.* 83 297–312. 10.1577/1548-8659(1953)83[297:IDOPS]2.0.CO;2

[B65] Rottmann R. W. Francis-Floyd R. Durborow R. (1992). *The Role of Stress in Fish Disease.* Stoneville, MS: Southern Regional Aquaculture Center, 474.

[B66] Salinas I. Díaz-Rosales P. Cuesta A. Meseguer J. Chabrillón M. Moriñigo M. (2006). Effect of heat-inactivated fish and non-fish derived probiotics on the innate immune parameters of a teleost fish (*Sparus aurata* L.). *Vet. Immunol. Immunopathol.* 111 279–286. 10.1016/j.vetimm.2006.01.020 16516307

[B67] Samal S. K. Das B. K. Pal B. B. (2014). Isolation, biochemical characterization, antibiotic susceptibility study of *Aeromonas hydrophila* isolated from freshwater fish. *Int. J. Curr. Microbiol. Appl. Sci.* 3 259–267.

[B68] Scharek L. Guth J. Filter M. Schmidt M. (2007). Impact of the probiotic bacteria *Enterococcus faecium* NCIMB 10415 (SF68) and *Bacillus cereus* var. toyoi NCIMB 40112 on the development of serum IgG and faecal IgA of sows and their piglets. *Arch. Anim. Nutr.* 61 223–234. 10.1080/17450390701431540 17760301

[B69] Sharon G. Segal D. Zilber-Rosenberg I. Rosenberg E. (2011). Symbiotic bacteria are responsible for diet-induced mating preference in *Drosophila melanogaster*, providing support for the hologenome concept of evolution. *Gut Microbes* 2 190–192. 10.4161/gmic.2.3.16103 21804354

[B70] Silva-Aciares F. Carvajal P. Mejías C. Riquelme C. (2010). Use of macroalgae supplemented with probiotics in the *Haliotis rufescens* (Swainson, 1822) culture in Northern Chile. *Aquacult. Res.* 42 953–961. 10.1111/j.1365-2109.2010.02678.x

[B71] Sissons J. (1989). Potential of probiotic organisms to prevent diarrhoea and promote digestion in farm animals – a review. *J. Sci. of Food Agric.* 49 1–13. 10.1002/jsfa.2740490102

[B72] Sugita H. Shibuya K. Shimooka H. Deguchi Y. (1996). Antibacterial abilities of intestinal bacteria in freshwater cultured fish. *Aquaculture* 145 195–203. 10.1016/s0044-8486(96)01319-1

[B73] Sullivan M. (2003). Active management of walleye fisheries in alberta: dilemmas of managing recovering fisheries. *N. Am. J. Fish. Manag.* 23 1343–1358. 10.1577/m01-232am

[B74] Suomalainen L. Tiirola M. Valtonen E. (2005). Influence of rearing conditions on *Flavobacterium columnare* infection of rainbow trout, *Oncorhynchus mykiss* (Walbaum). *J. Fish Dis.* 28 271–277. 10.1111/j.1365-2761.2005.00631.x 15892752

[B75] Sylvain F. Cheaib B. Llewellyn M. Gabriel Correia T. Barros Fagundes D. Luis Val A. (2016). pH drop impacts differentially skin and gut microbiota of the Amazonian fish tambaqui (*Colossoma macropomum*). *Sci. Rep.* 6:32032 10.1038/srep32032 PMC4989189 27535789

[B76] Tinh N. Dierckens K. Sorgeloos P. Bossier P. (2007). A review of the functionality of probiotics in the larviculture food chain. *Mar. Biotechnol.* 10 1–12. 10.1007/s10126-007-9054-9 18040740

[B77] Tremaroli V. Bäckhed F. (2012). Functional interactions between the gut microbiota and host metabolism. *Nature* 489 242–249. 10.1038/nature11552 22972297

[B78] Triyanto Kumamaru A. Wakabayashi H. (1999). The use of PCR Targeted 16S rDNA for Identification of genomovars of *Flavobacterium columnare*. *Fish Pathol.* 34 217–218. 10.3147/jsfp.34.217

[B79] Villamil L. Reyes C. Martínez-Silva M. (2012). In vivo and in vitro assessment of *Lactobacillus acidophilus* as probiotic for tilapia (*Oreochromis niloticus*, Perciformes:Cichlidae) culture improvement. *Aquacult. Res.* 45 1116–1125. 10.1111/are.12051

[B80] Vine N. Leukes W. Kaiser H. Daya S. Baxter J. Hecht T. (2004). Competition for attachment of aquaculture candidate probiotic and pathogenic bacteria on fish intestinal mucus. *J. Fish Dis.* 27 319–326. 10.1111/j.1365-2761.2004.00542.x 15189372

[B81] Welker T. L. Shoemaker C. A. Arias C. R. Klesius P. H. (2005). Transmission and detection of *Flavobacterium columnare* in channel catfish *Ictalurus punctatus*. *Dis. Aquat. Organ.* 63 129–138. 10.3354/dao063129 15819428

[B82] Wilson S. Nagler J. (2006). Age, but not salinity, affects the upper lethal temperature limits for juvenile walleye (*Sander vitreus*). *Aquaculture* 257 187–193. 10.1016/j.aquaculture.2005.10.045

[B83] Woo P. Cheung E. Leung K. Yuen K. (2001). Identification by 16S ribosomal RNA gene sequencing of an *Enterobacteriaceae* species with ambiguous biochemical profile from a renal transplant recipient. *Diagn. Microbiol. Infect. Dis.* 39 85–93. 10.1016/s0732-8893(01)00206-1 11248520

[B84] World Health Organization [WHO] (2014). *Antimicrobial Resistance. Fact Sheet N°194.* Available at: http://www.who.int/mediacentre/factsheets/fs194/en/ [accessed July 15, 2016].

[B85] Yan L. Boyd K. Grant Burgess J. (2002). Surface attachment induced production of antimicrobial compounds by marine epiphytic bacteria using modified roller bottle cultivation. *Mar. Biotechnol.* 4 356–366. 10.1007/s10126-002-0041-x 14961247

[B86] Zac Stephens W. Burns A. Stagaman K. Wong S. Rawls J. Guillemin K. (2015). The composition of the zebrafish intestinal microbial community varies across development. *ISME J.* 10 644–654. 10.1038/ismej.2015.140 26339860 PMC4817687

[B87] Zhou X. Tian Z. Wang Y. Li W. (2010). Effect of treatment with probiotics as water additives on tilapia (*Oreochromis niloticus*) growth performance and immune response. *Fish. Physiol. Biochem.* 36 501–509. 10.1007/s10695-009-9320-z 19363655

